# Eating dystonia in a case of neuroacanthocytosis

**Published:** 2015-01-05

**Authors:** Mohammad Rohani, Gholamali Shahidi

**Affiliations:** Department of Neurology, School of Medicine, Rasoul Akram Hospital, Iran University of Medical Sciences, Tehran, Iran

**Keywords:** Eating Dystonia, Neuroacanthocytosis, Video, Chorea, Movement Disorders

Neuroacanthocytosis is an autosomal recessive neurodegenerative disease, characterized by chorea, dementia, seizure, acanthocytes on peripheral blood smear and caudate atrophy on brain magnetic resonance imaging (MRI).^1^^,^^2^

These patients have severe orofacial dyskinesia and especially eating dystonia that causes severe eating problems and tongue and cheek biting. Eating or feeding dystonia, in combination with the above-mentioned signs and symptoms is characteristic of neuroacanthocytosis.^1^^-^^3^

Here, we present a video clip of a 40-year-old woman with typical eating dystonia .When she puts bolus in the mouth; dystonic movement of the tongue pushes it out (Video 1).

She had progressive choreiform movements especially orofacial dyskinesias since 10 years. Her brain MRI showed caudate atrophy and T2 and fluid-attenuated inversion recovery hyperintensity of caudate and putamens. On the peripheral blood smear, there were many acanthocytes.

Feeding dystonia is highly suggestive of neuroacanthocytosis and is a hallmark for this rare disease.^3^

## Notes

**How to cite this article:** Rohani M, Shahidi Gh. Eating dystonia in a case of neuroacanthocytosis. Iran J Neurol 2015; 14(1): 52.
